# Neutrophil Extracellular Traps at the Asthma + COPD Interface: Mechanisms, Experimental Models, and Therapeutic Targeting

**DOI:** 10.3390/cells15191769

**Published:** 2026-09-29

**Authors:** Leandro do Nascimento Camargo, Renato Fraga Righetti, Iolanda de Fátima Lopes Calvo Tibério

**Affiliations:** 1 Faculdade de Medicina FMUSP, Universidade de São Paulo, São Paulo 01246-903, Brazil; leandro.camargo@outlook.com.br (L.d.N.C.);; 2 Serviço de Reabilitação, Hospital Sírio-Libanês, São Paulo 01308-050, Brazil

**Keywords:** neutrophil extracellular traps, asthma, chronic obstructive pulmonary disease, neutrophilic inflammation, airway remodeling, animal models

## Abstract

**Highlights:**

**What are the main findings?**

- Neutrophil extracellular traps (NETs) emerge as a mechanistic link between neutrophilic inflammation, oxidative stress, epithelial injury, and extracellular matrix remodeling in the context of coexisting asthma and chronic obstructive pulmonary disease (COPD) features.
- Methodological heterogeneity in NET detection remains a major limitation, underscoring the need for multimodal approaches that integrate NET-associated molecular markers with imaging-based evidence of NET formation.

**What are the implications of the main findings?**

- Experimental models combining asthma- and COPD-like features are essential to determine whether NETs actively contribute to persistent inflammation and structural lung remodeling rather than merely reflect neutrophil activation.
- Therapeutic strategies targeting NET formation or persistence, particularly peptidylarginine deiminase 4 (PAD4) inhibition and deoxyribonuclease (DNase)-based approaches, warrant further mechanistic and translational investigation in the context of coexisting asthma and COPD features.

**Abstract:**

Asthma and chronic obstructive pulmonary disease (COPD) are distinct chronic airway diseases with different pathophysiological mechanisms, but they may coexist and share clinical, functional, inflammatory, and structural features. The historical terminology used to describe this coexistence has shifted away from the notion of a single overlap syndrome toward a traits-based framework, emphasizing coexisting clinical, functional, inflammatory, and structural features rather than a uniform disease entity. Within this context, neutrophil extracellular traps (NETs) are emerging as a potential mechanistic link between allergic airway inflammation, cigarette smoke injury, neutrophilic activation, mucus obstruction, and tissue remodeling. Animal models combining allergen exposure with cigarette smoke, lipopolysaccharide, infection, or elastase provide useful platforms to dissect specific components of the asthma-COPD interface. This review examines NET biology with a primary focus on animal models that reproduce overlapping asthma and COPD features, emphasizing how epithelial injury, oxidative stress, protease activity, neutrophilic inflammation, and inflammatory cytokine networks converge to promote NET formation. NETs, composed of extracellular deoxyribonucleic acid (DNA), citrullinated histone H3, myeloperoxidase, neutrophil elastase, and granular proteins, may amplify mucus viscosity, epithelial damage, innate immune activation, airway remodeling, emphysema-like injury, and persistent airflow limitation. Strategies to target this axis include deoxyribonuclease I (DNase I), peptidylarginine deiminase 4 (PAD4) inhibition, neutrophil elastase and myeloperoxidase blockade, antioxidant approaches, C-X-C motif chemokine receptor 2 (CXCR2) modulation, and interleukin-17 (IL-17) inhibition. These interventions act at distinct stages of neutrophil recruitment, NET formation, NET persistence, or NET-mediated tissue injury. A rigorous interpretation of these models requires precise terminology, multiparametric NET detection, and integration of lung mechanics, histology, inflammatory profiling, and structural outcomes. Targeting NETs may help shift preclinical research from broad overlap labels toward mechanism-driven therapeutic strategies for coexisting asthma and COPD features.

## 1. Introduction

Asthma and chronic obstructive pulmonary disease (COPD) are common chronic respiratory diseases characterized by airflow limitation and airway inflammation, although they differ in their predominant pathophysiological mechanisms and clinical manifestations. Asthma is typically characterized by variable respiratory symptoms and variable expiratory airflow limitation, frequently associated with type 2 airway inflammation, whereas COPD is characterized by persistent airflow obstruction resulting from airway and/or alveolar abnormalities and is commonly associated with chronic exposure to harmful particles or gases [1,2]. Despite these distinctions, some patients may present coexisting clinical, functional, inflammatory, and structural features of both diseases, creating important challenges for disease characterization and management [1,2,3].

### 1.1. Historical Evolution of the Concept of Asthma-COPD Overlap

The relationship between asthma and COPD has never been straightforward. For more than sixty years, it has moved between two competing interpretations: one emphasizing shared origins, the other insisting on diagnostic separation [4,5]. The Dutch Hypothesis, articulated in the 1960s and later revisited in the respiratory literature, proposed that asthma, chronic bronchitis, and emphysema might reflect different clinical expressions of a common predisposition, shaped by host-related factors and environmental exposures [4]. A different view soon gained influence. The British Hypothesis treated asthma and COPD as largely distinct disorders, emphasizing cigarette smoking, chronic mucus hypersecretion, respiratory infections, and progressive loss of lung function [5].

For decades, asthma and COPD were studied as separate diseases. Asthma studies commonly excluded smokers or individuals with substantial smoking exposure, while COPD trials often excluded patients with a previous diagnosis of asthma, atopy, allergic rhinitis, marked bronchodilator reversibility, or eosinophilic inflammation [1,2,3]. Patients carrying features of both diseases were common in daily practice, yet they were poorly represented in the evidence base that shaped therapeutic decisions [3,6].

Interest in this neglected group resurfaced in the early 2000s. The boundary between asthma and COPD was increasingly recognized as porous: some patients with asthma developed COPD-like traits over time, whereas patients with COPD could display asthma-like features such as bronchodilator reversibility, eosinophilia, atopy, or a prior history of asthma [6]. Gibson and Simpson later brought wider attention to the term overlap syndrome, particularly in older individuals with chronic obstructive airway disease who met clinical or functional criteria for both asthma and COPD [6]. In 2012, a Spanish consensus group proposed criteria for the mixed COPD-asthma phenotype, incorporating persistent airflow obstruction, previous asthma, marked bronchodilator response, sputum eosinophilia, elevated immunoglobulin E (IgE), and atopy [7]. Subsequent guidance from the Global Initiative for Asthma (GINA) and the Global Initiative for Chronic Obstructive Lung Disease (GOLD) used the overlap concept pragmatically while moving toward more descriptive approaches [1,2].

The problem was the word “syndrome”. It implied more than the data could support. A syndrome suggests a coherent entity, with a reproducible clinical profile, shared biology, and reasonably stable diagnostic criteria. Asthma-COPD overlap syndrome (ACOS) had none of these with sufficient consistency [2,3,8]. Patients labeled as ACOS, and later asthma-COPD overlap (ACO), were biologically diverse. Some had long-standing asthma with fixed airflow obstruction. Others had COPD with type 2 inflammation. Some had COPD superimposed on a previous history of asthma. Others reflected the cumulative effects of smoking, environmental exposure, airway remodeling, eosinophilic inflammation, and neutrophilic injury [2,3,8]. The label grouped them together, but it did not explain them. This is why the terminology changed. ACOS gradually became ACO, dropping the explicit claim of a “syndrome”. More recent guidance has moved even further, favoring descriptive language based on coexisting traits rather than a single diagnostic entity [1,2,8]. This historical shift, from a presumed overlap syndrome to a traits-based framework, is summarized in Figure 1. In that sense, “asthma-COPD overlap” is best treated as a historical and clinical shorthand, not as a disease with a fixed biological signature.

### 1.2. ACO: A New Disease, a Syndrome, or a Cluster of Coexisting Traits?

The central controversy around ACO is deceptively simple: what exactly is being named? A new disease? A syndrome? A clinical phenotype? Or merely the coexistence of traits that are already recognized within asthma and COPD? The literature has not settled this question. No universally accepted diagnostic definition exists, and studies have used markedly different entry criteria, including physician-diagnosed asthma, persistent airflow obstruction, bronchodilator reversibility, blood or sputum eosinophilia, atopy, elevated IgE, smoking exposure, age at symptom onset, and history of exacerbations [2,8,10,11,12]. The result is predictable: prevalence estimates vary widely, patient populations differ from one cohort to another, and comparisons across studies become fragile [12].

The term “overlap syndrome” was useful because it named a neglected clinical problem. It also carried conceptual baggage. A syndrome implies a recognizable constellation of features, some degree of biological coherence, and diagnostic boundaries that can be applied with reasonable reproducibility. ACO never fully met that standard [2,3,8]. Patients grouped under this label may follow very different biological paths. One patient may have childhood-onset asthma, decades of airway remodeling, and fixed airflow limitation. Another may have smoking-related COPD with blood eosinophilia and corticosteroid responsiveness. A third may have COPD superimposed on a previous asthma diagnosis. Others may reflect a more complex mixture of cigarette smoke, biomass exposure, chronic epithelial injury, type 2 inflammation, neutrophilic inflammation, mucus hypersecretion, and small-airway remodeling [2,3,8].

This heterogeneity is not a minor detail. It is the core of the problem. ACO is not a single mechanistic state. It is a clinical umbrella under which several phenotypes can be placed, sometimes for pragmatic reasons rather than biological precision. This explains why recent guideline language has become more cautious. GOLD no longer treats ACO as a distinct syndrome; instead, it emphasizes that asthma and COPD are different disorders that may coexist in the same individual and may share traits such as eosinophilia or partial bronchodilator reversibility [1,2]. GINA similarly stresses the need to identify asthma features in patients with persistent airflow limitation, because the presence of asthma changes treatment priorities, particularly the need for inhaled corticosteroid-containing therapy [2]. The direction is clear: less emphasis on a fixed label, more attention to measurable traits.

Genetic susceptibility adds another layer to this heterogeneity. Asthma and COPD have partially shared genetic determinants, suggesting that the coexistence of features from both diseases may arise from interactions between inherited susceptibility and environmental exposures rather than from a single disease-specific genetic program [13]. Genome-wide analyses of coexisting asthma and COPD have identified loci related to type 2 inflammation and fixed airflow obstruction, including *TSLP*, *IL17RD*, and *HLA-DQB1*, further supporting a spectrum of shared and disease-predominant genetic influences [13]. In COPD, transcriptomic and genetic analyses have also identified genes associated with neutrophilic inflammation, including *IL1B*, *CXCL2*, *CXCL3*, *PTGS2*, *SOD2*, and *TNFAIP3*, linking genetic and molecular heterogeneity to neutrophil-dominant inflammatory pathways [14].

A traits-based framework is more faithful to the data. It allows the clinician or investigator to ask sharper questions: Is there type 2 inflammation? Is airflow limitation fixed or variable? Is emphysema present? Is the disease driven by smoke, allergen exposure, infection, or a combination of insults? Are eosinophils, neutrophils, mucus plugging, oxidative stress, or epithelial injury dominant features? This approach aligns with the broader movement toward treatable traits and precision medicine in chronic airway disease, where phenotypes and endotypes are used to guide therapy rather than to defend rigid diagnostic borders [9].

### 1.3. Current Terminological Standardization

In this review, ACO will be used only in a historical sense or when referring to studies that explicitly adopted that label. For mechanistic and experimental discussion, we will use the term “animal models with overlapping asthma and COPD features.” This wording is deliberate. It avoids presenting ACO as a single disease entity and allows the discussion to stay anchored in measurable biology: phenotypes, endotypes, inflammatory patterns, structural abnormalities, and experimentally tractable mechanisms.

The field has moved in this direction for good reasons. Contemporary GOLD documents no longer frame ACO as a discrete syndrome; instead, they emphasize that asthma and COPD are different disorders that may coexist in the same individual and may share clinical or biological traits, such as eosinophilia or partial bronchodilator reversibility [1,2]. GINA takes a similarly pragmatic position. When asthma features are present in a patient with persistent airflow limitation, they must be recognized because they alter treatment priorities, particularly the need for inhaled corticosteroid-containing therapy to reduce the risk of severe exacerbations and asthma-related mortality [2].

This approach is reinforced by the 2026 GINA strategy, which uses “asthma + COPD” as a descriptive term for patients with features of both conditions rather than as a single disease entity. Importantly, when features or diagnoses of both asthma and COPD are present, GINA recommends treatment as asthma with an inhaled corticosteroid-containing regimen and advises against the use of long-acting β2-agonists (LABA) and/or long-acting muscarinic antagonists (LAMA) without inhaled corticosteroids (ICS) [2]. GOLD 2026 similarly states that patients with COPD and concomitant asthma should be treated according to asthma recommendations, with ICS forming an essential component of therapy [1].

The Allergic Rhinitis and its Impact on Asthma (ARIA) framework provides a complementary perspective rather than an alternative classification of asthma-COPD coexistence. ARIA emphasizes the interconnected nature of upper- and lower-airway inflammatory disease, and ARIA-endorsed recommendations on small-airway involvement recognize that the peripheral airways contribute importantly to the pathophysiology of both asthma and COPD. This broader airway perspective supports phenotypic characterization and individualized management according to the anatomical and inflammatory components involved [15].

### 1.4. Pathophysiological Rationale for the Asthma + COPD Association

Asthma and COPD meet at an inflammatory crossroads, but they do not arrive there by the same route. Asthma is classically driven by variable airflow limitation, airway hyperresponsiveness, mucus overproduction, and a type 2 immune program. This axis explains many canonical asthma features: eosinophilic inflammation, goblet cell metaplasia, airway smooth muscle contraction, mucus plugging, and steroid responsiveness. Yet asthma is not one disease. Severe asthma often escapes this tidy type 2 frame. Type 2-low and neutrophilic phenotypes may involve interleukin-17 (IL-17), epithelial injury, inflammasome activation, and relative corticosteroid insensitivity [16,17].

COPD is built differently. Chronic exposure to cigarette smoke, biomass, occupational particles, air pollution, or recurrent infection injures the airway and alveolar epithelium, creating a persistent inflammatory field dominated by oxidative stress, macrophage activation, neutrophilic influx, protease–antiprotease imbalance, mucus hypersecretion, small-airway remodeling, and alveolar wall destruction [1,18,19,20]. The major cellular actors include airway epithelial cells, alveolar macrophages, neutrophils, cluster of differentiation 8 (CD8)^+^ T cells, fibroblasts, and endothelial cells [18]. Together, these processes and defective repair contribute to extracellular matrix degradation, mucus obstruction, and progressive airflow limitation [18,19,20].

Cytokine and biomarker profiles may further refine this mechanistic distinction, although no single inflammatory marker reliably separates asthma from COPD. Type 2 inflammation, more commonly associated with asthma, is characterized by interleukin-4 (IL-4), interleukin-5 (IL-5), and interleukin-13 (IL-13) signaling and can be assessed clinically through blood or sputum eosinophils, fractional exhaled nitric oxide (FeNO), and IgE [2,21]. These biomarkers are particularly useful for inflammatory phenotyping and for predicting corticosteroid or biologic responsiveness [2,21,22]. In contrast, neutrophilic inflammatory patterns, frequently observed in COPD and in severe or type 2-low asthma, involve mediators such as interleukin-1β (IL-1β), interleukin-6 (IL-6), interleukin-8 (IL-8) also known as C-X-C motif chemokine ligand 8 (CXCL8), tumor necrosis factor-α (TNF-α), and IL-17, together with increased neutrophil recruitment and activation [14,16,22]. Importantly, these pathways are not disease-exclusive. Blood eosinophilia may also occur in COPD and can identify patients more likely to benefit from inhaled corticosteroid-containing therapy, whereas neutrophilic inflammation can occur in severe asthma [1,2,21]. Thus, cytokines and biomarkers are more informative as indicators of inflammatory endotypes and treatable traits than as stand-alone diagnostic markers distinguishing asthma from COPD [1,2,9,21].

The overlap between asthma and COPD is not merely the coexistence of symptoms. It is a collision of disease programs. In one trajectory, long-standing asthma leads to airway remodeling, mucus gland enlargement, smooth muscle hypertrophy, and fixed airflow obstruction. In another, COPD acquires asthma-like biology through eosinophilia, type 2 inflammation, bronchodilator reversibility, or steroid responsiveness [2,3,8]. A third pattern is more complex: allergic airway inflammation superimposed on cigarette smoke injury, infection, elastase-mediated tissue damage, or pollutant exposure. This is the space where eosinophils and neutrophils coexist, where mucus becomes persistent, where airway hyperresponsiveness and emphysema can appear in the same experimental system.

Animal studies make this convergence visible. Ovalbumin (OVA) models reproduce allergic sensitization, eosinophilic airway inflammation, type 2 cytokine production, mucus metaplasia, and airway hyperresponsiveness, while house dust mite (HDM)-based models provide complementary allergen-driven airway inflammation [8,23]. Cigarette smoke models reproduce several COPD-like features, including macrophage and neutrophil accumulation, oxidative stress, epithelial injury, mucus changes, and, with longer protocols, emphysema-like airspace enlargement [8,24]. Elastase models are cruder but powerful: they produce rapid airspace enlargement and protease-driven emphysema, making them useful when the question concerns matrix destruction, alveolar simplification, or emphysema-like injury [25]. Lipopolysaccharide (LPS) and infection models add another layer, mimicking neutrophilic exacerbation biology and innate immune amplification [8,24].

The newer overlap models are especially informative. In HDM + cigarette smoke models, smoke aggravates allergic airway inflammation, increases neutrophilic recruitment, worsens epithelial injury, disrupts tight-junction proteins such as zonula occludens-1 (ZO-1) and occludin, and enhances airway hyperresponsiveness [26]. OVA + LPS + smoke models produce mixed inflammatory signatures and broad transcriptomic remodeling, supporting their use as models of combined allergic, irritant, and innate immune activation [27]. OVA + porcine pancreatic elastase (PPE) or papain-based systems reproduce coexisting airway inflammation, airway hyperresponsiveness, emphysema-like changes, and altered lung mechanics [28,29]. More recently, HDM + elastase models have shown mixed granulocytic inflammation, expansion of monocyte-derived macrophages, neutrophils and eosinophils, increased *Mmp12*, *Il5*, and *Il13* gene expression, and simultaneous airway hyperreactivity, fibrosis, and emphysema; blockade of cluster of differentiation 131 (CD131) reduced inflammation and structural damage in this setting [30]. This is a useful signal: overlap biology may be coordinated by nodes that connect type 2 inflammation, macrophage activation, neutrophilic injury, and tissue remodeling.

In this inflammatory landscape, neutrophil extracellular traps (NETs) become biologically plausible rather than decorative. Neutrophils recruited by smoke, LPS, infection, IL-17, epithelial chemokines, and oxidative stress can release extracellular deoxyribonucleic acid (DNA) decorated with citrullinated histone H3, myeloperoxidase (MPO), neutrophil elastase, and other granular proteins [31,32,33,34]. NETs may contribute to mucus alteration, epithelial injury, extracellular protease activity, and inflammatory amplification [8,17,32,34]. In severe asthma, extracellular DNA and NET-associated signals have been linked to inflammasome activation and disease severity [17]. In COPD, NET burden has been associated with airflow limitation, disease severity, and exacerbation-related inflammation [35,36,37]. The overlap setting contains several NET-promoting triggers at once: allergen-driven epithelial activation, cigarette smoke-derived reactive oxygen species (ROS), infection or LPS, neutrophilic chemokines, and protease injury.

The pathophysiological rationale for asthma + COPD, then, is not that one disease simply “adds” to the other. The interaction is nonlinear. Type 2 inflammation can increase mucus and epithelial vulnerability; smoke can shift the inflammatory tone toward neutrophils and oxidative stress; infection can activate innate immunity; elastase and matrix metalloproteinases (MMPs) can damage matrix and alveolar architecture; NETs may further amplify inflammatory and tissue-injury loops [1,2,3,8,17,26,27,29,30,31,35,36,37,38,39]. The final phenotype may include eosinophilia, neutrophilia, mucus plugging, epithelial barrier failure, airway remodeling, emphysema, exacerbation susceptibility, reduced steroid responsiveness, and persistent airflow limitation.

Together, these interacting inflammatory and structural programs provide the pathophysiological basis for studying NETs as a potential amplification mechanism at the asthma–COPD interface.

### 1.5. Animal Models as Tools to Study Overlapping Asthma and COPD Features

Animal models are indispensable when the question is causal. They allow timing, dose, tissue sampling, pathway inhibition, and invasive physiology in a way that clinical studies cannot. Still, they have a hard ceiling: no mouse, rat, or guinea pig model reproduces the full clinical heterogeneity of human asthma, COPD, or asthma + COPD. A preclinical study should therefore not claim to generate “an ACO model” as though ACO were a single entity waiting to be reproduced in mice. A more rigorous strategy is to model defined components of the asthma-COPD interface: allergic airway inflammation, cigarette smoke exposure, eosinophilia, neutrophilic inflammation, mucus hypersecretion, epithelial injury, oxidative stress, airway remodeling, emphysema, and persistent airflow limitation. For this reason, the expression “animal models with overlapping asthma and COPD features” is more precise than “ACO models”. It recognizes the heterogeneity of the human condition and the inherently partial nature of animal modeling, while keeping the mechanistic question clear.

Classical asthma models based on OVA or HDM reproduce selected features of allergic airway disease, including eosinophilic inflammation, type 2 cytokine production, mucus metaplasia, and airway hyperresponsiveness [8,23]. HDM-based models also provide a clinically relevant allergen-driven stimulus. These systems are powerful, but incomplete. Many protocols remain acute or subacute, heavily type 2-biased, and poorly suited to capture fixed airflow limitation, emphysema, chronic epithelial injury, or the neutrophil-rich biology seen in severe asthma and COPD-like disease.

COPD models occupy a different experimental terrain. Chronic cigarette smoke exposure remains an etiologically relevant approach for tobacco-related COPD, inducing macrophage and neutrophil accumulation, oxidative stress, epithelial damage, mucus changes, small-airway pathology, and, with longer protocols, emphysema-like airspace enlargement [8,24]. Elastase instillation is faster and cleaner mechanistically. It drives protease-mediated alveolar destruction and emphysema, but it does not recapitulate the slow, toxic, inflammatory ecology of cigarette smoke exposure [25]. LPS, bacterial products, viral infection, particulate matter, and ozone are used in different preclinical settings to enhance innate immune activation, neutrophilic inflammation, exacerbation-like responses, or pollutant-driven injury [8,24].

For overlapping asthma and COPD features, combined models are the most informative. OVA or HDM plus cigarette smoke can place allergic inflammation, mucus production, and airway hyperresponsiveness into the same lung environment as oxidative stress, epithelial injury, and neutrophilic inflammation [8,26]. Allergen plus LPS, with or without cigarette smoke, enriches the neutrophilic and innate immune component, producing mixed inflammatory patterns [8,27]. Allergen plus elastase creates a different construct: allergic airway disease superimposed on emphysema-like alveolar injury [28,30,38]. These are not interchangeable “ACO models.” They are mechanistic approximations of specific asthma + COPD traits. Figure 2 summarizes how combined experimental exposures reproduce selected asthma- and COPD-related traits and converge on epithelial injury, oxidative stress, neutrophil recruitment, NET formation, structural remodeling, emphysema-like injury, and altered airflow.

Recent animal work has made this point increasingly clear. In an HDM + cigarette smoke model, smoke aggravated allergic airway disease, increased bronchoalveolar lavage fluid (BALF) neutrophils, worsened lung injury, enhanced airway hyperresponsiveness, and disrupted epithelial tight-junction proteins, including ZO-1 and occludin [26]. In OVA + LPS + smoke models, transcriptomic profiling revealed a broad inflammatory program, with increased leukocyte infiltration and multiple inflammatory mediators [27]. OVA + PPE and papain-based models have also reproduced coexisting asthma-like and COPD-like traits, including airway inflammation, emphysema-like changes, altered lung mechanics, and airway hyperresponsiveness [28,29].

Camargo et al. [38] used a BALB/c mouse model combining OVA-induced allergic inflammation with porcine pancreatic elastase-induced emphysema-like injury, then tested whether IL-17 blockade could modulate the overlap response. The signal was important: anti-IL-17 reduced respiratory system resistance, elastance, airway resistance, tissue mechanics abnormalities, and inflammatory cells in BALF in the overlap model. That finding positions IL-17 as a functional inflammatory amplifier in this experimental asthma + COPD-like setting. It also provides a therapeutic bridge toward NET biology, because IL-17-driven chemokine networks can contribute to neutrophil recruitment and downstream tissue injury.

The newest overlap models are becoming more refined. A 2025 HDM + elastase study showed that combined allergic and emphysema-like injury produced airway hyperreactivity, emphysema, mixed granulocytic inflammation, expansion of monocyte-derived macrophages, neutrophils, eosinophils, increased *Mmp12*, *Il5*, and *Il13* gene expression, and pathway enrichment for type 2 inflammation and macrophage activation [30]. Blocking CD131 reduced inflammation, airway hyperreactivity, fibrosis, and emphysema [30]. This is the direction the field is moving: away from labels, toward nodes that coordinate several pathological programs at once.

Despite their mechanistic value, mouse models have important translational limitations. Species-specific differences in airway anatomy, lung physiology, immune responses, and susceptibility to pulmonary insults limit direct extrapolation to humans [41,42]. Moreover, many experimental protocols reproduce disease features over days or weeks, whereas human asthma and COPD develop over years or decades under the influence of repeated environmental exposures, aging, genetic susceptibility, and comorbidities [41,42]. Experimental challenges such as OVA or HDM sensitization, cigarette smoke, LPS, and elastase can reproduce selected inflammatory or structural components of human disease but cannot capture its full clinical heterogeneity [8,24,41,42]. Therefore, these models should be interpreted as tools for investigating defined mechanisms and overlapping traits rather than as complete replicas of human asthma + COPD [41,42].

### 1.6. NETs as a Common Mechanistic Axis

NETs are not simply debris from dying neutrophils. They are structured extracellular scaffolds composed of decondensed chromatin decorated with histones, MPO, neutrophil elastase (NE), and other granular proteins [8,32,43]. Brinkmann and colleagues first described NETs as an antimicrobial mechanism by which neutrophils immobilize and kill microbes outside the cell [32]. That original idea still holds. Yet NETs are also recognized as potentially tissue-damaging inflammatory structures when excessively formed or insufficiently cleared [8,17,32,35,36,40].

Although this review focuses specifically on neutrophil extracellular traps, extracellular trap formation is not restricted to neutrophils. Eosinophils can release eosinophil extracellular traps (EETs), which have been implicated in severe asthma and may contribute to airway inflammation and mucus abnormalities [44,45]. Extracellular trap formation has also been described in other immune cell populations, including macrophages [46]. This distinction is particularly relevant at the asthma–COPD interface, where eosinophilic and neutrophilic inflammatory pathways may coexist. Extracellular traps derived from different cellular sources could therefore contribute to the extracellular DNA burden, epithelial injury, and amplification of local inflammation. Importantly, these structures should not collectively be termed NETs; rather, NETs represent the neutrophil-derived component of the broader extracellular trap response.

The biology is intricate. In canonical NET formation, neutrophils can be activated by microbial products, immune complexes, crystals, cytokines, oxidant stress, LPS, or other inflammatory signals. Reduced nicotinamide adenine dinucleotide phosphate (NADPH) oxidase-derived ROS participate in some NET-forming pathways, while NE and MPO contribute to chromatin remodeling and NET formation [8,34,43]. Peptidylarginine deiminase 4 (PAD4) can citrullinate histones, weakening histone–DNA interactions and favoring chromatin decondensation [33]. The molecular sequence is context-dependent rather than universal, and different NET-forming pathways may depend on distinct combinations of ROS, PAD4, and granular enzymes [33,34,43].

Autophagy has also been implicated in the regulation of NET formation. In human neutrophils, experimental inhibition of autophagy has been shown to impair chromatin decondensation and NET formation under selected conditions, while autophagy can interact with ROS-dependent signaling during this process [47]. The mechanistic target of rapamycin (mTOR)-autophagy pathway may also regulate NET release, further supporting a functional interaction between autophagic signaling and NET formation [48]. Moreover, autophagy-dependent mechanisms have been implicated in NET formation in inflammatory and fibrotic contexts [49,50]. However, the contribution of autophagy appears to depend on the stimulus and experimental context and should not be interpreted as a universal requirement for all NET-forming pathways.

There are other routes. Not all NETs are generated through the same program. Some forms are PAD4-dependent; others are less reliant on PAD4. Some require NADPH oxidase-derived ROS; others appear ROS-independent. Mitochondrial DNA can contribute to extracellular trap structures under selected conditions, and vital NET release has also been described [51,52]. This heterogeneity matters because it complicates interpretation. An increase in extracellular DNA does not necessarily indicate NETosis, and citrullinated histone H3 (citH3) alone is insufficient to confirm NET formation. MPO–DNA or NE–DNA complexes may provide greater specificity; however, these markers should ideally be interpreted alongside imaging evidence demonstrating the colocalization of extracellular DNA with neutrophil-derived proteins [52,53].

For this reason, NET assessment should be multiparametric. A robust experimental approach combines biochemical assays and morphology: extracellular DNA, MPO-DNA complexes, NE-DNA complexes, citH3, neutrophil elastase, MPO, and immunofluorescence or confocal microscopy demonstrating extracellular DNA structures associated with neutrophils [40,53]. Tissue context is essential. In inflamed lungs, DNA can also derive from necrotic epithelial cells, macrophages, eosinophils, or other dying cells. NETs must be demonstrated rather than inferred from a single biomarker.

In asthma, NETs have been linked to severe or neutrophilic disease and to specific experimental inflammatory contexts [17,31]. Radermecker and colleagues showed that locally instructed neutrophils expressing high levels of C-X-C motif chemokine receptor 4 (CXCR4) can trigger environment-driven allergic asthma through NET formation, connecting neutrophil programming and airway inflammation in an experimental setting [31]. Lachowicz-Scroggins and colleagues reported extracellular DNA, NET-associated signals, and inflammasome activation in severe asthma, supporting an association between NET-related biology and disease severity [17]. Additional evidence suggests that NETs may also contribute to structural remodeling. Chrysanthopoulou and colleagues demonstrated that NETs generated in response to fibrosis-related stimuli, including cigarette smoke, promoted lung fibroblast activation and differentiation toward a myofibroblast phenotype, with increased connective tissue growth factor expression and collagen production [50]. More recently, Ntinopoulou and colleagues reported increased NET formation associated with interleukin-17A (IL-17A) during childhood asthma exacerbation and showed that IL-17A-enriched NETs promoted a pre-fibrotic phenotype in lung fibroblasts, including increased cellular communication network factor 2 (CCN2) protein expression, migratory activity, and collagen release [54]. Together, these findings support a potential NET/IL-17A/fibroblast axis linking neutrophilic inflammation to structural remodeling. Mechanistically, NET-associated components may contribute to epithelial injury and inflammatory amplification, but the strength of evidence differs across mechanisms and should not be interpreted as proof that NETs drive asthma pathogenesis.

In COPD, evidence for enhanced NET formation is substantial. NETs have been detected in the airways of patients with stable and exacerbated COPD and have been associated with airflow limitation, neutrophilic inflammation, and clinical disease severity [35,36,40]. Dicker and colleagues demonstrated that increased sputum NET-associated complexes were associated with more severe COPD and lower lung function, while NET burden was also related to airway microbiota diversity and impaired bacterial phagocytosis [36].

Cigarette smoke and the inflammatory microenvironment of COPD provide multiple conditions capable of favoring NET formation, including oxidative stress, epithelial injury, protease activity, persistent innate immune activation, and altered neutrophil antimicrobial function [8,19,35,36,40]. Infectious stimuli may further amplify this response during exacerbations. Katsoulis and colleagues provided experimental and human evidence linking NETs to virus-induced COPD exacerbations [37]. Candidate NET-associated readouts include extracellular or cell-free DNA, DNA–protein complexes such as MPO–DNA or NE–DNA, citrullinated histone H3 (citH3), NE, MPO, and PAD4-related signals. However, these markers differ substantially in specificity, and neither extracellular DNA nor any single NET-associated biomarker should be considered definitive evidence of NET formation in isolation; whenever feasible, biochemical measurements should be integrated with imaging and cellular context [40,53]. These human observations, candidate NET-associated biomarkers, and their potential pathological consequences are summarized in Figure 3.

This is where asthma + COPD becomes especially interesting. The overlap setting brings together many of the strongest NET-inducing conditions: allergen-driven epithelial activation, cigarette smoke-derived ROS, LPS or infection, IL-17-associated neutrophil recruitment, elastase-mediated injury, mucus hypersecretion, and defective epithelial repair. The lung becomes permissive. Type 2 inflammation can expand mucus-producing pathways and epithelial vulnerability; smoke and infection recruit neutrophils; IL-17 and chemokine gradients pull them into the airway wall and lumen; oxidative stress lowers the threshold for NET formation. Once released, NETs may act as a molecular bridge linking neutrophilic inflammation to mucus plugging, epithelial injury, remodeling, emphysema-like damage, and reduced treatment responsiveness.

The most plausible model is not that NETs “cause” the entire asthma + COPD phenotype. That would be too broad. Rather, NETs may amplify a specific inflammatory circuit within overlapping disease features. In this circuit, activated neutrophils release extracellular DNA, citH3, MPO, and NE; these structures increase mucus viscosity, injure the epithelium, activate innate immune pathways, and feed back into IL-1β, tumor necrosis factor (TNF), IL-17, C-X-C motif chemokine ligand 1 (CXCL1)/C-X-C motif chemokine ligand 2 (CXCL2), and macrophage–neutrophil signaling. The consequence is not merely inflammation. It is persistent, spatially organized injury: mucus retention in the lumen, epithelial barrier failure, proteolytic stress in the airway wall, remodeling in the submucosa, and impaired lung mechanics.

### 1.7. Why NETs Matter in Asthma + COPD Models

A review focused on NETs and NET-blocking strategies in animal models with overlapping asthma and COPD features is timely for three reasons. The first is conceptual. Clinical terminology has moved away from ACOS/ACO as a fixed diagnostic entity and toward a traits-based description of coexisting asthma and COPD features, yet the experimental literature has not fully absorbed this shift [1,2,3,8,9]. Many preclinical studies still use broad labels where the biology would be better described in sharper terms: allergen-driven inflammation, cigarette smoke injury, elastase-mediated emphysema, LPS-induced neutrophilic inflammation, epithelial barrier disruption, mucus hypersecretion, or altered lung mechanics. Precision begins with naming.

The second reason is methodological. Combined animal models are increasingly used to approximate the asthma-COPD interface, but they are not interchangeable. Allergen + cigarette smoke, allergen + LPS, allergen + elastase, and allergen + smoke + infection each generate a different inflammatory architecture [8,26,27,28,29]. One model may emphasize type 2 inflammation with epithelial injury. Another may enrich IL-17-dependent neutrophilia. A third may foreground protease-driven alveolar destruction. Treating all of them as “ACO models” obscures the experimental signal. A better approach is to ask what each model actually reproduces, which cells dominate, which mediators rise, which structural lesions appear, and which therapeutic node is being tested.

The third reason is mechanistic. NETs sit at a biologically plausible intersection between asthma-like and COPD-like pathology. They connect neutrophil recruitment, oxidative stress, infection or LPS exposure, cigarette smoke injury, protease activity, epithelial damage, and tissue remodeling [8,17,26,27,29,30,31,32,35,36,37,40]. This makes them attractive not because they explain everything, but because they may explain a particularly destructive inflammatory circuit: the point at which neutrophils stop being transient responders and become contributors to persistent airway injury.

NET-targeted interventions can act at several levels. Some reduce neutrophil recruitment, including strategies directed at C-X-C motif chemokine receptor 2 (CXCR2) or IL-17 and related chemokine networks [8,38]. Others interfere with intracellular NET-forming machinery, including ROS-dependent pathways, MPO, NE, and PAD4-dependent histone citrullination [33,34,43]. Deoxyribonuclease I (DNase I) works downstream by digesting extracellular DNA and dismantling NET scaffolds, but the evidence for DNase-based approaches in chronic airway disease remains largely preclinical; the nanozyme example in the reference list is from bowel inflammation rather than asthma or COPD [55]. PAD4 inhibition is a more proximal strategy for investigating PAD4-dependent NET formation, and Lewis et al. showed that pharmacological PAD4 inhibition can disrupt NET formation in mouse and human neutrophils [56]. MPO or NE inhibition may influence NET formation and/or NET-associated tissue injury, but these effects are pathway and context-dependent [34]. Newer approaches, including DNase-based and nanomaterial platforms, remain experimental and should not be presented as established therapies for asthma + COPD [55].

These therapeutic opportunities span multiple levels of the NET axis, from upstream modulation of epithelial and inflammatory signals to inhibition of neutrophil recruitment and activation, interference with intracellular NET-forming machinery, degradation of extracellular NET scaffolds, and neutralization of NET-associated effector molecules (Figure 4).

These distinctions matter. Blocking NETs is not one intervention. It is a family of strategies aimed at different points in the neutrophil response: recruitment, priming, chromatin decondensation, extracellular DNA persistence, and granular-protein toxicity. A study using DNase I asks a different question from one using anti-IL-17, PAD4 inhibition, neutrophil elastase blockade, or CXCR2 antagonism. Lumping them together would flatten the biology.

The objective of this review is to critically examine the role of NETs in animal models that reproduce overlapping asthma and COPD features, with particular attention to model design, inflammatory phenotype, NET detection methods, and therapeutic blockade strategies. The review will not treat ACO as a single disease. Instead, it will map how defined experimental insults—including allergen exposure, cigarette smoke, LPS or infection, elastase, oxidative stress, and type 2/neutrophilic inflammation—converge on NET formation and downstream airway-parenchymal injury. The goal is to identify where NETs are merely associated with inflammation, where they appear mechanistically relevant, and where NET-targeted blockade may offer a rational path for future translational studies.

## 2. Clinical Implications

### 2.1. NETs and NET-Associated Components as Biomarkers of Disease Activity

The clinical value of studying NETs in overlapping asthma and COPD features lies in precision, not in relabeling patients. The ACO label has always been too broad to guide therapy on its own. A patient with long-standing asthma, fixed airflow limitation, eosinophilia, and mucus plugging is not the same as a patient with smoking-related emphysema, recurrent infections, neutrophilic sputum, and partial bronchodilator reversibility. Both may be placed under an overlap umbrella.

Their biology may be very different. NETs could help separate some of these trajectories. If validated in clinical cohorts, NET-associated markers such as extracellular DNA, MPO-DNA complexes, NE-DNA complexes, citH3, neutrophil elastase, and related protease signatures may identify patients in whom neutrophilic inflammation is not merely present but mechanistically active. That distinction matters. A neutrophil count alone tells us who is in the airway. NETs may tell us what those neutrophils are doing.

NETs may also explain why some patients remain symptomatic despite apparently appropriate therapy. Extracellular DNA and protease-rich NET structures can increase mucus viscosity, damage epithelial barriers, and sustain innate immune activation. That biology is poorly addressed by standard bronchodilation alone. It may also contribute to corticosteroid insensitivity, particularly in mixed eosinophilic–neutrophilic disease.

For patients with overlapping asthma and COPD features, the most realistic future is not a single “anti-NET therapy.” It is stratified intervention. NET biomarkers may become part of a broader inflammatory panel that includes blood and sputum eosinophils, neutrophils, FeNO, IgE, exacerbation history, microbiome patterns, imaging, mucus burden, and lung function. In that setting, NETs could help identify a subgroup in whom neutrophil-driven tissue injury, mucus plugging, and remodeling are central rather than incidental.

The clinical message is direct: move from labels to mechanisms. “Asthma + COPD” should not be treated as a final diagnosis, but as a prompt to ask better questions. Which traits dominate? Which inflammatory circuits are active? Is the airway injury type 2-high, neutrophilic, infectious, protease-driven, NET-high, or some unstable combination of these? The answer may determine whether NETs are just a biomarker of severity or a target worth pursuing.

### 2.2. NETs as Potential Therapeutic Targets

This has therapeutic consequences. Patients with dominant type 2 inflammation may benefit from inhaled corticosteroids or biologics targeting IL-5, interleukin-4 receptor α (IL-4Rα), IgE, or epithelial alarmins. For patients with neutrophil-rich, NET-high disease, potential strategies under investigation include: reduction in neutrophil recruitment, control of infection, attenuation of oxidative stress, inhibition of neutrophil elastase or MPO, DNase-based mucus modulation, or blockade of upstream pathways such as IL-17/CXCR2 in carefully selected contexts. The target should follow the trait.

There is a caution. NET blockade is not automatically benign. NETs participate in host defense, especially against bacteria and fungi. DNase may reduce mucus obstruction, but it may not neutralize histones, elastase, or MPO. PAD4 inhibition may reduce one NET-forming pathway while leaving others intact. Neutrophil elastase inhibition may protect tissue but could alter antimicrobial responses. Clinical translation will require careful phenotyping, infection monitoring, and endpoints that go beyond symptom scores.

## 3. Future Perspectives

Studying NETs in animal models with overlapping asthma and COPD features gives the field a way out of the conceptual trap created by the ACO label. ACO has never behaved like a single disease. It is uneven, shifting, and biologically plural. Experimental models should not pretend otherwise. The most informative systems are not those that claim to reproduce “ACO” in full, but those that isolate tractable disease traits: allergic airway inflammation, cigarette smoke injury, eosinophilia, neutrophilic recruitment, epithelial barrier failure, mucus hypersecretion, elastase-driven emphysema, airway remodeling, altered lung mechanics, and persistent airflow limitation.

In that setting, NETs become more than an inflammatory by-product. They occupy a dangerous intersection. Smoke-derived oxidative stress, infection or LPS exposure, IL-17-driven neutrophilia, protease activity, mucus plugging, epithelial injury, and tissue remodeling all converge on neutrophil activation. Once released, NET components—including extracellular DNA, citH3, MPO, neutrophil elastase, histones, and granular proteins—can thicken secretions, damage epithelial surfaces, activate innate immune pathways, and feed persistent inflammatory loops. They may therefore be more than passive readouts, acting as plausible amplifiers.

The field now needs discipline. Extracellular DNA alone should not be treated as proof of NET formation. NET detection should combine biochemical readouts, such as MPO-DNA, NE-DNA, citH3, extracellular DNA, and neutrophil elastase, with imaging evidence showing extracellular DNA colocalized with neutrophil granule proteins. The same standard should apply to model selection. HDM + smoke, OVA + smoke, allergen + LPS, and allergen + elastase models do not ask the same question. Each reproduces a different fragment of the asthma-COPD interface. Calling all of them “ACO models” flattens the biology and weakens interpretation.

Therapeutic studies need equal precision. DNase I, PAD4 inhibitors, neutrophil elastase inhibitors, MPO inhibitors, antioxidants, anti-CXCR2 approaches, and IL-17 blockade act at different points in the neutrophil-NET axis. Some limit recruitment. Some alter priming. Some interfere with chromatin decondensation. Some dismantle preformed NET scaffolds. Others blunt protease-driven tissue injury. The strongest experiments will compare these strategies directly, pair them with lung mechanics and structural outcomes, and ask whether NET reduction changes disease biology rather than simply lowering inflammatory signal intensity.

Several questions remain unresolved. Are NETs active drivers of airway remodeling and emphysema-like damage, or mostly footprints of neutrophil-rich inflammation? Do they help explain corticosteroid insensitivity in mixed asthma + COPD models? Is IL-17 upstream of NET formation in this context, or part of a reciprocal amplification loop? Can DNase reduce mucus obstruction without compromising antimicrobial defense? Are PAD4-independent pathways dominant in smoke- or infection-driven lung injury? These are not abstract questions. They are experimentally accessible.

The next generation of models should be chronic, layered, and closer to human disease trajectories. Allergen exposure combined with cigarette smoke, elastase, LPS, infection, aging, or pollutant exposure will likely be more informative than short, single-hit protocols. Sex as a biological variable should be built in. So should time. Lung mechanics, histology, BALF cytology, single-cell profiling, spatial imaging, microbiome analysis, and NET-specific assays can then be integrated to define which cellular circuits matter, where they operate, and when they become targetable.

## 4. Conclusions

NETs should not be considered a universal explanation for asthma + COPD. Rather, current evidence supports their role as a defined and potentially targetable inflammatory axis within overlapping disease features, shaped by neutrophilic inflammation, epithelial injury, oxidative stress, protease activity, mucus abnormalities, and tissue remodeling. Experimental models organized around defined traits rather than diagnostic labels will be essential to determine whether NETs are primarily biomarkers of neutrophil-rich inflammation or mechanistic amplifiers that can be therapeutically targeted at the asthma-COPD interface.

## Figures and Tables

**Figure 1:**
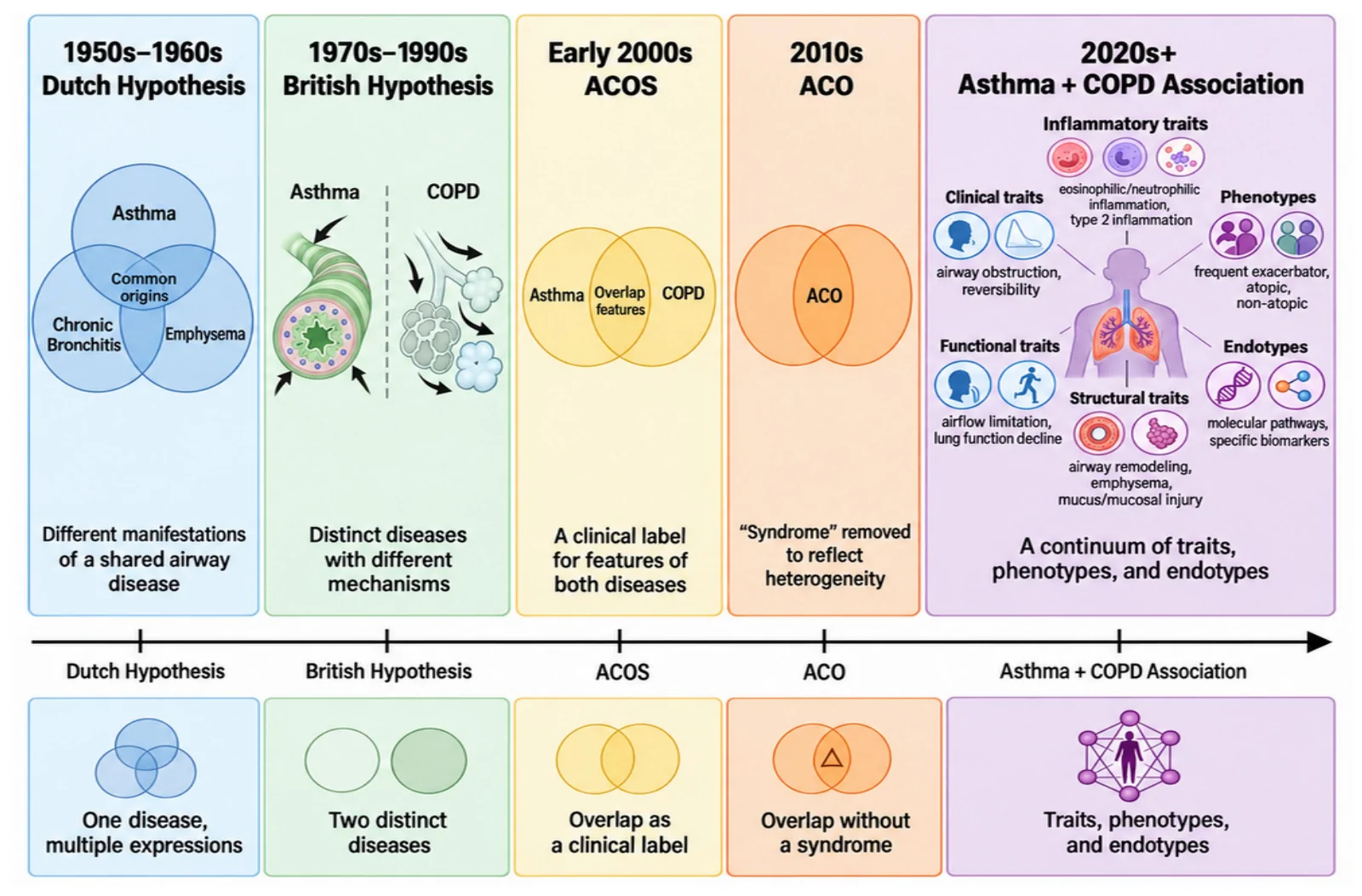
Historical evolution of the terminology and conceptual framework of asthma–COPD association. Legend: evolution of the concepts used to describe the coexistence of asthma and chronic obstructive pulmonary disease (COPD). The Dutch Hypothesis proposed shared origins among chronic obstructive airway diseases, whereas the British Hypothesis emphasized asthma and COPD as distinct disorders. The subsequent concepts of asthma–COPD overlap syndrome (ACOS) and asthma–COPD overlap (ACO) attempted to describe patients presenting features of both diseases. More recently, the field has moved away from considering ACO as a single disease entity toward a multidimensional framework based on coexisting clinical, functional, inflammatory, and structural traits, phenotypes, and endotypes. This transition supports characterization of the asthma + COPD association through treatable traits rather than a fixed overlap syndrome [1,2,3,9]. The horizontal arrow indicates the chronological progression of these conceptual frameworks from left to right. In the British Hypothesis panel, inward-pointing arrows schematically indicate airway narrowing, whereas outward-pointing arrows indicate airspace enlargement.

**Figure 2:**
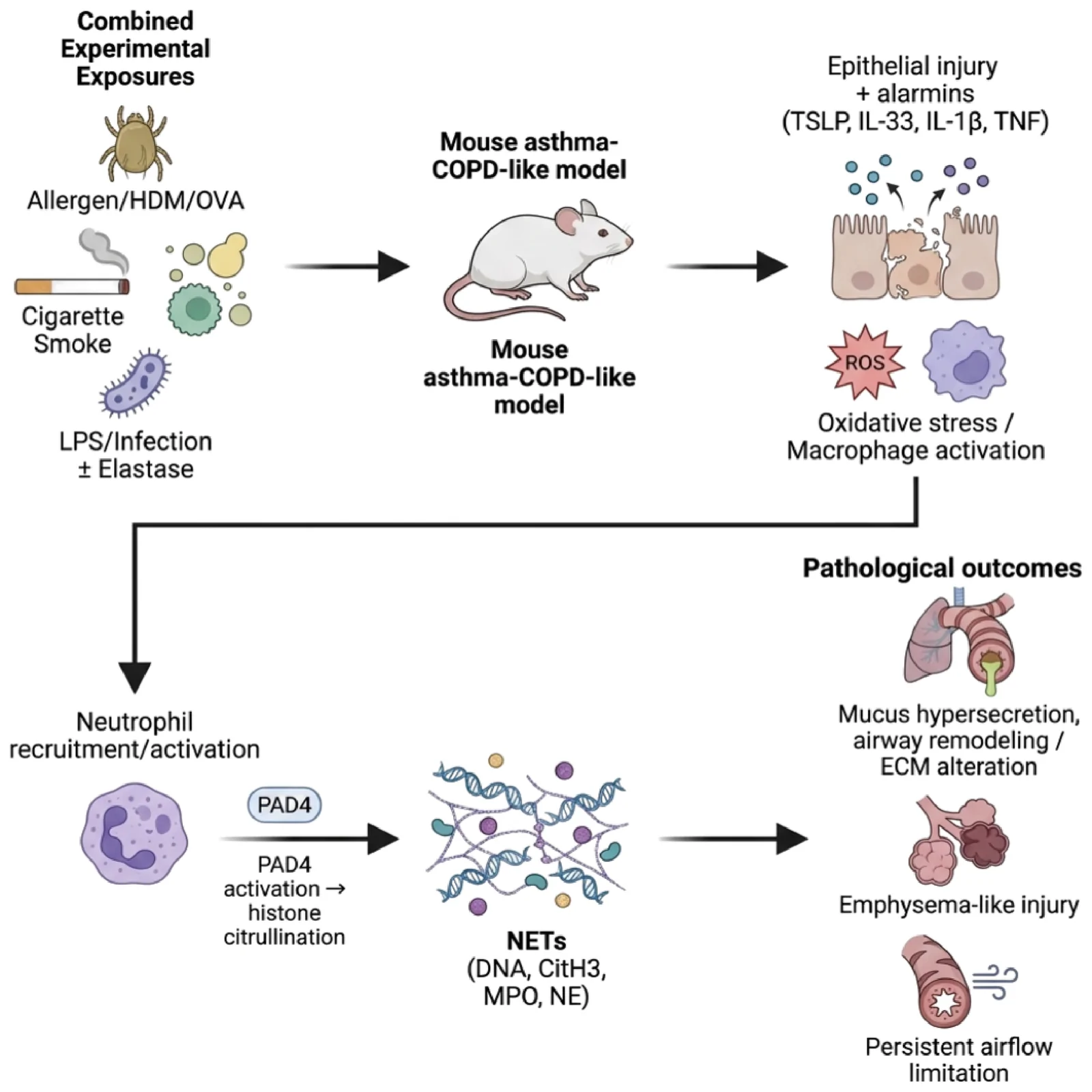
Experimental models of overlapping asthma and COPD features and their convergence on NET-associated lung injury. Legend: Experimental models combining allergic stimuli, such as ovalbumin (OVA) or house dust mite (HDM), with cigarette smoke, lipopolysaccharide (LPS), infection, or elastase reproduce selected asthma- and chronic obstructive pulmonary disease (COPD)-related traits rather than a single asthma–COPD overlap (ACO) entity. These exposures generate variable combinations of type 2 and neutrophilic inflammation, epithelial injury, oxidative stress, mucus hypersecretion, airway hyperresponsiveness, remodeling, and emphysema-like injury [8,26,27,28,29,30]. Neutrophil activation may promote peptidylarginine deiminase 4 (PAD4)-dependent histone citrullination and neutrophil extracellular trap (NET) formation, releasing extracellular deoxyribonucleic acid (DNA) associated with citrullinated histone H3 (citH3), myeloperoxidase (MPO), and neutrophil elastase (NE) [32,33,34]. NET-associated inflammation may subsequently amplify epithelial and extracellular matrix (ECM) injury, remodeling, and functional impairment, providing a mechanistic framework for testing the causal contribution of the NET axis [17,35,36,37,39,40]. Arrows indicate the proposed sequence from combined experimental exposures to epithelial injury and inflammatory activation in the upper row, continuing through neutrophil recruitment and activation, NET formation, and potential pathological outcomes in the lower row. Colors are schematic and distinguish illustrated structures and components; they do not represent quantitative values or a specific molecular color code. Additional abbreviations: TSLP, thymic stromal lymphopoietin; IL-33, interleukin-33; IL-1β, interleukin-1β; TNF, tumor necrosis factor; ROS, reactive oxygen species.

**Figure 3:**
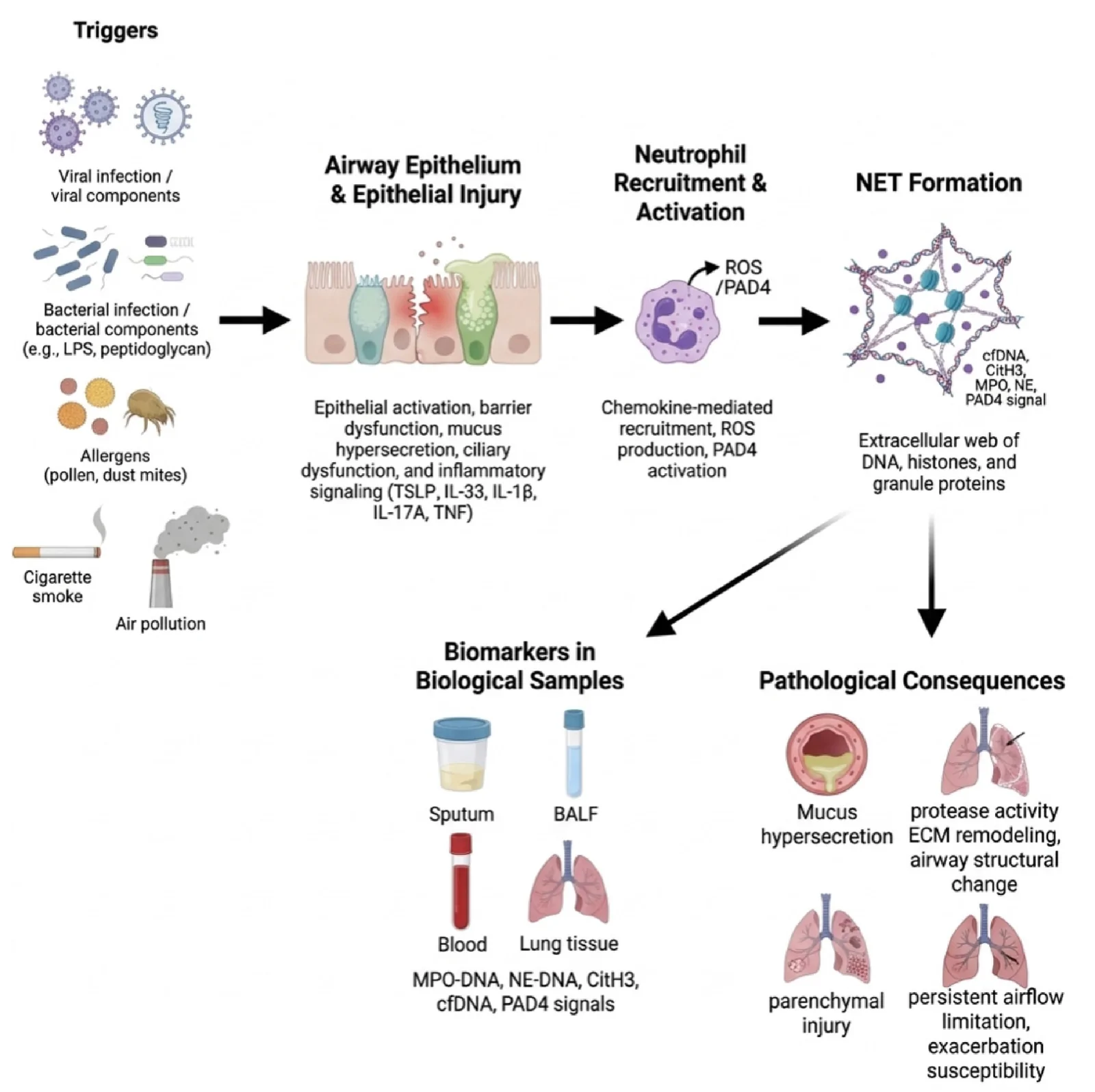
Neutrophil extracellular trap (NET)-associated inflammation in human asthma and chronic obstructive pulmonary disease (COPD): triggers, candidate biomarkers, and pathological consequences. Legend: Environmental and disease-related stimuli, including allergens, cigarette smoke, air pollution, viral infection or viral components, and bacterial infection or bacterial components (e.g., lipopolysaccharide (LPS) and peptidoglycan), may promote epithelial activation and injury, inflammatory signaling, neutrophil recruitment and activation, and NET formation in human airway disease. Inflammatory mediators associated with these processes include interleukin-1β (IL-1β), interleukin-17A (IL-17A), interleukin-33 (IL-33), thymic stromal lymphopoietin (TSLP), and tumor necrosis factor (TNF). Neutrophil activation and reactive oxygen species (ROS)- and peptidylarginine deiminase 4 (PAD4)-associated pathways may contribute to NET formation, resulting in extracellular structures composed of deoxyribonucleic acid (DNA), histones, and neutrophil granule proteins. Candidate NET-associated readouts in sputum, bronchoalveolar lavage fluid (BALF), blood, and lung tissue include extracellular or cell-free DNA (cfDNA), myeloperoxidase (MPO)–DNA and neutrophil elastase (NE)–DNA complexes, citrullinated histone H3 (citH3), MPO, NE, and PAD4-related signals. Increased NET-associated signals have been reported in severe asthma and in stable and exacerbated COPD and have been associated with inflammation, disease severity, impaired lung function, and exacerbations [17,31,35,36,37,40]. Potential pathological consequences include mucus hypersecretion, protease activity, extracellular matrix (ECM) remodeling, airway structural changes, parenchymal injury, persistent airflow limitation, and increased susceptibility to exacerbations. These NET-associated readouts remain predominantly research biomarkers, and no single marker should be considered definitive evidence of NET formation in isolation [40,53]. Beige/pink cells represent the airway epithelium, with red shading indicating epithelial injury. The lilac cell with a multilobed purple nucleus represents a neutrophil. The extracellular filamentous mesh depicts a NET composed of DNA and associated proteins; colors within this mesh do not distinguish individual molecular components.

**Figure 4:**
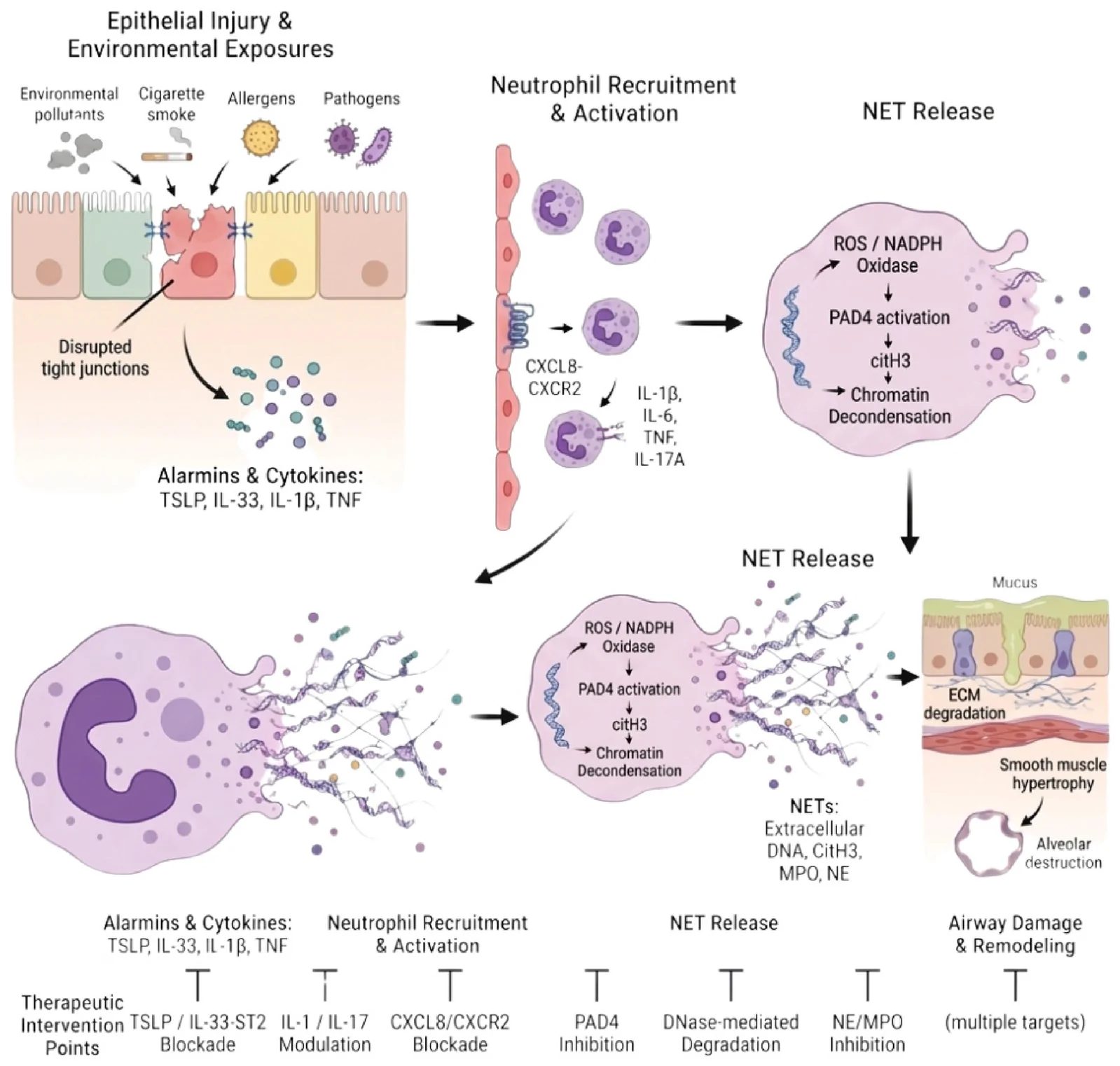
Therapeutic targeting of the neutrophil–NET axis: potential intervention points and future strategies. Legend: Potential therapeutic interventions can target different stages of the inflammatory cascade leading to neutrophil extracellular trap (NET) formation and NET-mediated tissue injury. Upstream approaches include modulation of epithelial and inflammatory pathways, such as interleukin-17 (IL-17), whereas the C-X-C motif chemokine ligand 8 (CXCL8)–C-X-C motif chemokine receptor 2 (CXCR2) axis represents a potential target for neutrophil recruitment [8,38]. NET formation may be modulated through pathways involving reactive oxygen species (ROS)/reduced nicotinamide adenine dinucleotide phosphate (NADPH) oxidase, myeloperoxidase (MPO), neutrophil elastase (NE), and peptidylarginine deiminase 4 (PAD4)-dependent histone citrullination [33,34,43]. Direct experimental PAD4 inhibition, including compounds such as GSK484, represents a strategy to investigate PAD4-dependent NET formation [56]. Downstream approaches include deoxyribonuclease (DNase)-mediated degradation of extracellular deoxyribonucleic acid (DNA) and inhibition of NET-associated effectors such as NE and MPO [34,55]. These approaches are at different stages of development, and direct PAD4/NET-targeted therapies remain predominantly preclinical in chronic airway disease. The multicolored epithelial cell layer represents the airway epithelium, with red shading highlighting epithelial injury. Lilac cells with multilobed purple nuclei represent neutrophils. The extracellular filamentous mesh represents released NETs; its colors do not identify individual molecular components. In the airway damage and remodeling panel, the green layer represents mucus, and the red spindle-shaped cells represent airway smooth muscle. Arrows indicate the proposed sequence of events, and T-shaped bars mark potential therapeutic intervention points. Additional abbreviations: TSLP, thymic stromal lymphopoietin; IL-33, interleukin-33; IL-1β, interleukin-1β; IL-1, interleukin-1; IL-6, interleukin-6; IL-17A, interleukin-17A; TNF, tumor necrosis factor; ST2, suppression of tumorigenicity 2, a receptor for IL-33; citH3, citrullinated histone H3; ECM, extracellular matrix.

## Data Availability

No new datasets were generated or analyzed in this study. Data sharing is not applicable to this article.
